# Supplementary material for: Modeling glioblastoma heterogeneity as a dynamic network of cell states
Source: Mol Syst Biol. 2021 Sep 16;17(9):e10105. doi: 10.15252/msb.202010105 (PMC8444284; doi:10.15252/msb.202010105)
Supplement: Supplementary file 6 — Source Data for Figure 5 [file MSB-17-e10105-s004.zip › Figure5A_sourcedata/GSEA_3017/hallmarks_stateD.GseaPreranked.1621934542601/HALLMARK_P53_PATHWAY.html]

Details for gene set HALLMARK\_P53\_PATHWAY[GSEA]

|  || Dataset | state23017 |
| Phenotype | NoPhenotypeAvailable |
| Upregulated in class | na\_neg |
| GeneSet | HALLMARK\_P53\_PATHWAY |
| Enrichment Score (ES) | -0.29858294 |
| Normalized Enrichment Score (NES) | -1.2657812 |
| Nominal p-value | 0.19509476 |
| FDR q-value | 0.3023157 |
| FWER p-Value | 1.0 |
Table: GSEA Results Summary

  

Fig 1: Enrichment plot: HALLMARK\_P53\_PATHWAY      
 Profile of the Running ES Score & Positions of GeneSet Members on the Rank Ordered List

  

| PROBE | GENE SYMBOL | GENE\_TITLE | RANK IN GENE LIST | RANK METRIC SCORE | RUNNING ES | CORE ENRICHMENT || 1 | TM4SF1 |  |  | 17 | 0.464 | 0.0224 | No |
| 2 | ZFP36L1 |  |  | 29 | 0.409 | 0.0452 | No |
| 3 | BTG1 |  |  | 50 | 0.350 | 0.0566 | No |
| 4 | RPL18 |  |  | 87 | 0.302 | 0.0517 | No |
| 5 | RPS12 |  |  | 129 | 0.271 | 0.0407 | No |
| 6 | PPP1R15A |  |  | 149 | 0.259 | 0.0457 | No |
| 7 | PPM1D |  |  | 162 | -0.251 | 0.0556 | No |
| 8 | EPHX1 |  |  | 216 | -0.255 | 0.0341 | No |
| 9 | SDC1 |  |  | 300 | -0.264 | -0.0102 | No |
| 10 | PMM1 |  |  | 309 | -0.264 | 0.0039 | No |
| 11 | CYFIP2 |  |  | 334 | -0.267 | 0.0057 | No |
| 12 | XPC |  |  | 362 | -0.269 | 0.0054 | No |
| 13 | POLH |  |  | 467 | -0.282 | -0.0538 | No |
| 14 | FBXW7 |  |  | 494 | -0.284 | -0.0522 | No |
| 15 | SLC3A2 |  |  | 517 | -0.287 | -0.0473 | No |
| 16 | APP |  |  | 626 | -0.306 | -0.1077 | No |
| 17 | RAD51C |  |  | 670 | -0.313 | -0.1171 | No |
| 18 | FDXR |  |  | 703 | -0.320 | -0.1174 | No |
| 19 | TM7SF3 |  |  | 704 | -0.320 | -0.0928 | No |
| 20 | PDGFA |  |  | 949 | -0.395 | -0.2520 | No |
| 21 | CCP110 |  |  | 1010 | -0.420 | -0.2664 | Yes |
| 22 | PLXNB2 |  |  | 1015 | -0.423 | -0.2371 | Yes |
| 23 | BAX |  |  | 1032 | -0.434 | -0.2162 | Yes |
| 24 | DDB2 |  |  | 1045 | -0.443 | -0.1915 | Yes |
| 25 | PLK2 |  |  | 1047 | -0.444 | -0.1582 | Yes |
| 26 | UPP1 |  |  | 1101 | -0.469 | -0.1634 | Yes |
| 27 | SAT1 |  |  | 1109 | -0.473 | -0.1326 | Yes |
| 28 | HSPA4L |  |  | 1131 | -0.497 | -0.1108 | Yes |
| 29 | MDM2 |  |  | 1135 | -0.499 | -0.0749 | Yes |
| 30 | PHLDA3 |  |  | 1137 | -0.501 | -0.0373 | Yes |
| 31 | PCNA |  |  | 1142 | -0.504 | -0.0018 | Yes |
| 32 | ZMAT3 |  |  | 1269 | -0.759 | -0.0414 | Yes |
| 33 | CDKN1A |  |  | 1306 | -1.046 | 0.0109 | Yes |
Table: GSEA details [plain text format]

  

Fig 2: HALLMARK\_P53\_PATHWAY: Random ES distribution      
 Gene set null distribution of ES for **HALLMARK\_P53\_PATHWAY**
